# Postoperative outcomes in minimally invasive total versus supracervical hysterectomy for endometriosis: a NSQIP study

**DOI:** 10.1007/s00404-024-07749-y

**Published:** 2024-10-16

**Authors:** Raanan Meyer, Jill McDonnell, Kacey M. Hamilton, Rebecca J. Schneyer, Gabriel Levin, Kelly N. Wright, Matthew T. Siedhoff

**Affiliations:** 1 Division of Minimally Invasive Gynecologic Surgery, Department of Obstetrics and Gynecology, Cedars Sinai Medical Center, Los Angeles, CA USA; 2 The Dr, Pinchas Bornstein Talpiot Medical Leadership Program, Sheba Medical Center, Tel Hashomer, Ramat-Gan, Israel; 3 Department of Obstetrics and Gynecology, Swedish Medical Center, Seattle, WA USA; 4 Lady Davis Institute for Cancer Research, Jewish General Hospital, McGill University, Quebec, Canada

**Keywords:** Complications, Pelvic pain, Laparoscopy, Robotic surgery

## Abstract

**Purpose:**

To study the rate and odds of 30 day postoperative complications among patients undergoing minimally invasive total (TLH) compared to supracervical (LSCH) hysterectomy for endometriosis.

**Study design:**

A cohort study of patients with a diagnosis of endometriosis undergoing hysterectomy. We used prospectively collected data from the American College of Surgeons National Surgical Quality Improvement Program database from 2012 to 2020. We compared short-term (30 day) complications, following minimally invasive TLH and LSCH for endometriosis. The primary outcome was the risk of any postoperative complications according to the surgical approach.

**Results:**

A total of 5,278 patients were included, 4,952 (93.8%) underwent TLH and 326 (6.2%) underwent LSCH. The incidence of any complication was significantly lower in the LSCH group compared to the TLH group (3.7% vs. 8.5%, *p* = .001). Both major complications (1.5% vs. 3.7%, *p* = 0.043) and minor complications (2.8% vs. 5.4%, *p* = .039) were less frequent in the LSCH group compared to the TLH group.

In multivariable regression analysis, patients undergoing LSCH had significantly lower odds of any complication [aOR 95%CI 0.40 (0.22–0.72)], and of minor complications [aOR 95%CI 0.47 (0.24–0.92)] compared to TLH.

**Conclusions:**

LSCH is associated with a lower odd of short-term postoperative complications compared to TLH for patients with endometriosis.

**Supplementary Information:**

The online version contains supplementary material available at 10.1007/s00404-024-07749-y.

## What does this study add to the clinical work?

**Table:** 

Among women undergoing minimally invasive hysterectomy for endometriosis, supracervical hysterectomy is associated with a lower odd of short-term postoperative complications when compared to total hysterectomy. This study sheds light on an understudied topic, and can be used for preoperative counseling and shared decision-making.	

## Introduction

Endometriosis is caused by the presence of endometrial-like glands and stroma lesions outside the uterine cavity. Among reproductive-age women, endometriosis prevalence is 10–15% [1]. Symptoms include dysmenorrhea, chronic pain, dyspareunia, dysuria, dyschezia, infertility, decrease in quality in life, and significant financial burden [2–5]. Surgical resection of endometriosis is offered to women with symptoms refractory to medical therapy, or for deep infiltrating endometriosis, and include conservative and definitive options. When possible, a minimally invasive approach is usually preferred [5–7]. Definitive surgery includes hysterectomy, with or without oophorectomy, and may be pursued for women who do not plan future childbearing and have failed other treatment options [8].

minimally invasive hysterectomy options include total laparoscopic (tlh) and laparoscopic supracervical (lsch) hysterectomies, where the cervix is not removed. compared with tlh, lsch is associated with slightly shorter surgical times, less blood loss during surgery, and less post-operative fever and urinary retention, but with higher risk of cyclical vaginal bleeding [9]. the european society of human reproduction and embryology (eshre) recommends performing tlhs for endometriosis in a practice guideline from 2022 [5]. however, this recommendation is based on experts’ opinion and is not supported by strong evidence. currently, data regarding the short-term impact of tlh compared to lsch for endometriosis are scant.

We sought to study the association between minimally invasive hysterectomy type and the risk of short-term postoperative complications, using the American College of Surgeons National Surgical Quality Improvement Program (NSQIP) database.

## Materials and methods

### Study design

This was a cohort study of data from the NSQIP database. We included women who underwent minimally invasive hysterectomy, laparoscopic or robotic, for endometriosis, as diagnosed post-operatively, between the years 2012 and 2020.

The NSQIP database is a large, comprehensive database, including records from approximately 700 participating hospitals. Data are collected prospectively, and include preoperative, intraoperative and 30 day postoperative variables. Participating hospitals have trained and certified Surgical Clinical Reviewers that collect variables in a variety of methods. The NSQIP database has been well described and validated [10, 11]. Details on data collection process and variables definitions can be found on the NSQIP website (https://www.facs.org/quality-programs/acs-nsqip).

We compared the outcomes of TLH and LSCH. The primary outcome was the occurrence of any or major postoperative complications by hysterectomy type. Secondary outcomes were the different types of complications included.

### Patient population

We identified patients through Current Procedural Terminology (CPT) codes, as presented in Table S1. We used the International Classification of Diseases Ninth/Tenth edition (ICD-9/10) codes to identify women with endometriosis, presented in Table S2. There is a single postoperative ICD code provided per case in the NSQIP database, corresponding to the condition recorded as the postoperative diagnosis in the brief operative note, operative report, and/or after the return of the pathology reports [11].

Our cohort included both conventional and robot-assisted minimally invasive hysterectomies. Both approaches have the same CPT code and are not reported separately in the NSQIP database. We excluded vaginal-only hysterectomies as this approach may limit inspection of the peritoneum and disease extent, and as severe endometriosis is a relative contraindication to vaginal hysterectomy [7, 12–14]. We also excluded laparoscopic-assisted vaginal hysterectomies, which are inconsistently defined and may vary based on the amount of the procedure performed vaginally [12]. We further excluded non-elective surgeries, cases with malignancy, and cases with pre-operative sepsis. Cases with ICD-9 code 617.0 and ICD-10 code N80.0- endometriosis of uterus were excluded as well, as those may have represented adenomyosis without pelvic disease, as well as ICD-9 code 617.6 and ICD-10 code 80.6- endometriosis in scar of skin, indicating non-peritoneal disease.

### Data collection

We collected baseline, preoperative and intraoperative characteristics, and postoperative complications. Baseline and preoperative characteristics included the following: age, body mass index (BMI), use of tobacco, diabetes mellitus, hypertension treated with medications, chronic obstructive pulmonary disease, immunosuppressive therapy, bleeding disorders, and American Society of Anesthesiologists (ASA) physical status system class. Surgical characteristics included uterine weight (defined by CPT codes as above or below 250 g), concomitant procedures performed during hysterectomy, identified based on CPT codes, total operative time and hospital length of stay.

We classified postoperative complications as major or minor using the Clavien–Dindo classification system [15]. This validated classification grades complications from 1 to 5, with grades 3–5 considered major complications. Major complications, occurring within 30 days of surgery, included: organ space surgical-site infection, deep incisional surgical-site infection, wound dehiscence, cerebrovascular accident, pulmonary embolism, deep venous thromboembolism, cardiac arrest, myocardial infarction, reoperation, or death. Minor post-operative complications included blood transfusion (within 72 h of surgery start time), superficial surgical-site infection, urinary tract infection, acute renal insufficiency, and pneumonia.

### Statistics analysis

Categorical variables were analyzed using the Chi-square test and Fisher’s exact test as appropriate. Student’s t-test was used to compare continuous variables. Categorical variables were reported as proportions and continuous variables as mean (standard deviation). Multivariable logistic regression analyses were performed to identify variables independently associated with any, major, and minor postoperative complications by hysterectomy type. The multivariable logistic regression analysis models included factors that were statistically significantly different in the univariate analysis and were considered clinically relevant. Results are reported as adjusted odds ratio (aOR) and 95% confidence interval (CI). A 2-sided p value < 0.05 was considered statistically significant. Statistical analyses were performed using Software Package for Statistics and Simulation (IBM SPSS version 27, IBM Corp, Armonk, NY) and R [ Core Team (2021)].

#### Ethical approval

As the data used for this study are publicly available and do not include protected health information, the Cedars-Sinai Institutional Review Board provided a Letter of Exemption concluding that approval is not required.

## Results

A total of 5,278 women met inclusion criteria during the study period, 4,952 (93.8%) TLH and 326 (6.2%) LSCH. Baseline characteristics are presented in Table 1. Subjects in the TLH group were younger and more likely to be smokers (*p* < 0.001 and *p* = 0.044 respectively). White women underwent more TLHs than LSCHs. Other baseline characteristics were comparable between groups.

**Table 1:** Baseline and operative characteristics of women undergoing minimally invasive total or supracervical hysterectomy for endometriosis

Characteristics	Total laparoscopic/robotic hysterectomy (*n* = 4,952)	Laparoscopic/robotic supracervical hysterectomy (*n* = 326)	*p* value
Age, years	39.5 (7.2)	41.0 (6.7)	< 0.001
Race			
White	3816 (77.1)^a^	220 (67.5)^b^	< 0.001
Black or African American	290 (5.9)^a^	19 (5.8)^a^	
Asian	176 (3.6)^a^	12 (3.7)^a^	
American Indian or Alaska Native	40 (0.8)^a^	4 (1.2)^a^	
Native Hawaiian or Pacific Islander	22 (0.4)^a^	1 (0.3)^a^	
Other or unknown	608 (12.3)^a^	70 (21.5)^b^	
Hispanic ethnicity	366 (8.4)	19 (7.4)	0.643
Body mass index, mean, kg/m2	29.7 (7.4)	30.1 (7.7)	0.446
Tobacco use	905 (18.3)	45 (13.8)	0.044
Diabetes mellitus	208 (4.2)	15 (4.6)	0.671
Hypertension	697 (14.1)	52 (16.0)	0.367
Chronic obstructive pulmonary disease	16 (0.3)	0 (0.0)	0.620
Immunosuppressive therapy	75 (1.5)	2 (0.6)	0.237
Bleeding disorders	28 (0.6)	3 (0.9)	0.437
Preoperative hematocrit value, %	28.6 (37.6)	29.3 (36.4)	0.738
Preoperative blood transfusion	2 (0.0)	1 (0.3)	0.174
ASA classification III/IV	811 (16.4)	61 (18.8)	0.280

Data are n (%) or mean (Standard deviation)

*ASA* American society of anesthesiologists

Each subscript letter denotes a subset of groups’ categories whose column proportions do not differ significantly from each other at the .05 level

Uterine weight > 250 g rate was comparable between groups as well as having concomitant procedures performed (Table 2). Fulguration/excision of ovarian lesions, pelvic viscera, or peritoneal surface and appendectomy were more common in the TLH group (*p* = 0.008 and *p* = 0.034 respectively). Total operative time was comparable between groups (135.8 min for TLH vs. 140.9 min for LSCH, *p* = 0.199).

**Table 2:** Surgical characteristics of women undergoing minimally invasive total or supracervical hysterectomy for endometriosis

	Total laparoscopic/robotic hysterectomy (*n* = 4,952)	Laparoscopic/robotic supracervical hysterectomy (*n* = 326)	*p* value
Uterine weight > 250 g	343 (6.9)	25 (7.7)	0.575
Concomitant procedures performed during hysterectomy			
Fulguration/excision of ovarian lesions, pelvic viscera, or peritoneal surface	942 (19.0)	43 (13.2)	0.008
Lysis of adhesions	481 (9.7)	36 (11.0)	0.441
Bladder procedure	57 (1.2)	3 (0.9)	>0 .999
Ureter procedure	156 (3.2)	5 (1.5)	0.131
Ureterolysis	115 (2.3)	5 (1.5)	0.445
Appendectomy	142 (2.9)	3 (0.9)	0.034
Intestinal surgery with enterotomy	85 (1.7)	9 (2.8)	0.188
Intestinal surgery without enterotomy	323 (6.5)	26 (8.0)	0.300
Ovarian cystectomy/drainage	14 (0.3)	0 (0.0)	>0 .999
Colporrhaphy	47 (0.9)	2 (0.6)	0.768
Colpopexy or vaginopexy	86 (1.7)	5 (1.5)	>0 .999
Contaminated or dirty/Infected wound class	68 (1.4)	3 (0.9)	0.801
Total operative time, minutes	135.8 (69.5)	140.9 (67.8)	0.199
Hospital Length of stay, days	0.9 (5.8)	0.6 (5.6)	0.318

Data are n (%) or mean (Standard deviation)

Complications occurred in 8.5% of TLH and 3.7% of LSCH cases, *p* = 0.001 (Table 3). Major complications occurred in 3.7% and 1.5% of TLH and LSCH cases (*p* = 0.043), and minor complications in 5.4% and 2.8% (*p* = 0.039). Urinary tract infection rate was higher after TLHs (3.0% vs. 0.9%, *p* = 0.025). No other single major or minor complications differed significantly between groups.

**Table 3:** Postoperative characteristics among women undergoing minimally invasive total or supracervical hysterectomy for endometriosis

Characteristics	Total laparoscopic/robotic hysterectomy (*n* = 4,952)	Laparoscopic/robotic supracervical hysterectomy (*n* = 326)	*p* value
Any complication	420 (8.5)	12 (3.7)	0.001
Major complication	182 (3.7)	5 (1.5)	0.043
Minor complication	266 (5.4)	9 (2.8)	0.039
Readmission	171 (3.5)	5 (1.5)	0.077
Minor complications			
Transfusion	46 (0.9)	2 (0.6)	0.768
Superficial surgical-site infection	74 (1.5)	3 (0.9)	0.630
Urinary tract infection	149 (3.0)	3 (0.9)	0.025
Renal insufficiency	0 (0.0)	0 (0.0)	NA
Pneumonia	6 (0.1)	1 (0.3)	0.360
Major complications			
Organ space surgical-site infection	87 (1.8)	3 (0.9)	0.374
Deep incisional surgical-site infection	6 (0.1)	0 (0.0)	>0 .999
Wound dehiscence	14 (0.3)	1 (0.3)	0.616
Pulmonary embolism	11 (0.2)	0 (0.0)	>0 .999
Cardiac arrest	0 (0.0)	0 (0.0)	NA
Myocardial infarction	10 (0.2)	0 (0.0)	0.504
Cerebrovascular accident	1 (0.0)	0 (0.0)	>0 .999
Deep venous thromboembolism	10 (0.2)	1 (0.3)	0.504
Ventilation > 48 h	0 (0.0)	0 (0.0)	NA
Sepsis	23 (0.5)	1 (0.3)	>0 .999
Septic shock	0 (0.0)	0 (0.0)	NA
Reoperation	73 (1.5)	2 (0.6)	0.327
Reintubation	2 (0.0)	0 (0.0)	>0 .999
Progressive renal insufficiency	4 (0.1)	0 (0.0)	> 0.999
Death	0 (0.0)	0 (0.0)	NA

Data are n (%)

In the multivariable logistic regression analysis of factors associated with complications (Table 4), LSCH was independently associated with a lower risk of any complications compared to TLH [adjusted odds ratio (aOR) 95% confidence interval (CI) 0.40 (0.22–0.72), *p* = 0.002]. Major complications were not independently associated with type of hysterectomy [aOR 95% CI 0.44 (0.18–1.07), *p* = 0.070 for LSCH compared with TLH]. Minor complications were associated with type of hysterectomy [aOR 95% CI 0.47 (0.24–0.92), *p* = 0.029 for LSCH compared with TLH].

**Table 4:** Multivariable regression analysis of factors associated with complications

	Any complications* aOR(95% CI)	*p* value	Major complications** (aOR, 95% CI)	*p* value	Minor complications*** (aOR, 95% CI)	*p* value
Total laparoscopic hysterectomy	Reference		Reference		Reference	
Laparoscopic Supracervical hysterectomy	0.40 (0.22–0.72)	0.002	0.44 (0.18–1.07)	0.070	0.47 (0.24–0.92)	0.029

*Adjusted for: race, ASA classification III/IV, peritoneal, adnexal or intestinal surgery without enterotomy, lysis of adhesions, enterotomy, colporrhaphy or pexy

**Adjusted for: age, race, smoking, peritoneal, adnexal or intestinal surgery without enterotomy, lysis of adhesions, enterotomy

***Adjusted for: race, diabetes mellitus, chronic obstructive pulmonary disease, immunosuppressive therapy, chronic hypertension, ASA classification III/IV, adnexal or intestinal surgery without enterotomy, lysis of adhesions, enterotomy, colporrhaphy or pexy

## Discussion

In patients with endometriosis, LSCH was associated with lower odds of any or minor complications when compared to TLH in multivariable regression analysis. Difference was driven primarily by higher proportions of UTI in the TLH group. There was no difference in major complications between methods.

Supracervical hysterectomy may be preferred by surgeons for reduced operating times, technical ease, and lower blood loss [9, 16]. However, it poses additional concerns for patients with endometriosis and some experts have advised caution in a supracervical approach. Complete excision of endometriosis may be less likely with supracervical amputation, especially if the presence of microscopic endometriosis in the cervical stump cannot be excluded [16]. In addition, retained endometrial tissue from the cervical stump has been theorized to increase risk of recurrence through retrograde menstruation [16]. Approximately 5–10 percent of patients will have persistent cyclic bleeding after supracervical hysterectomy, though the literature is mixed on whether endometriosis increases that risk [17, 18]. In a case–control study of 17 women who underwent LSCH, a history of endometriosis was associated with an increased risk of subsequent trachelectomy [19]. In addition, morcellation of the uterine body has also been thought to increase the risk of recurrence or new-onset disease through abdominal seeding [16].

Only one case–control study has examined the incidence of endometriosis after supracervical hysterectomy, but their findings lacked statistical significance and were deemed inconclusive [20]. A single-blinded randomized control trial of women with dysmenorrhea found no difference in self-reported dysmenorrhea at 12 months or patient satisfaction between total and supracervical hysterectomy groups [21]. However, that study was limited by small sample size (*n* = 31 in each treatment arm), and most patients did not have endometriosis detected during surgery. No randomized controlled trials have examined post-operative complications, symptoms or disease recurrence specifically in an endometriosis population, and systematic reviews on disease or symptom recurrence have shown mixed results [22].

The findings of our study can be compared to a 2012 Cochrane Review studying total and supracervical hysterectomy for benign gynecologic conditions [9]. That review found no differences between the two groups in short, intermediate, and long-term outcomes and complications. However, the authors’ primary outcomes were largely based on symptoms and quality of life, which is in contrast to our focus on complications as defined by the Clavien–Dindo classifications [9]. In a 2019 meta-analysis that did examine short-term outcomes in benign conditions, no difference was found between total and supracervical laparoscopic hysterectomy in rates of blood transfusion, urinary tract injury, febrile morbidity, and readmission [23]. In contrast to these studies, we did find fewer short-term complications with supracervical hysterectomy. This difference may be attributable to our focus only on an endometriosis population, whose surgeries have been shown to have more inherent complication risk compared to other benign indications [24]. A 2023 European multicenter study used Clavien–Dindo to analyze short-term surgical complications of TLH in endometriosis, and their rates of minor and major complications after TLH were very similar to our findings [25]. Clavien–Dindo has also previously been used in a NSQIP study evaluating the increase in morbidity in hysterectomies for endometriosis compared to other benign conditions, though approach of hysterectomy was not examined closely [24].

The primary strength of this study is its use of the NSQIP database for identifying complications. This large nationwide, prospective database across many medical systems increases the generalizability of our results. The large sample size allowed the analysis of rare complications. Using the Clavien–Dindo classification system also allows for a more standardized approach to examining adverse outcomes and comparing our findings to existing studies. Endometriosis is common, though the literature examining surgical approach and adverse outcomes in this population is lacking. With our focus on endometriosis, this work adds an important piece to the literature regarding an overall safety comparison of two very common gynecologic surgeries in an understudied population.

Our study has several limitations. Though NSQIP data collection is prospective, this study is observational. This study was not powered to detect differences between TLH and LSCH approaches for rare complications. The NSQIP database only includes one ICD code for each case, so it is possible that not all cases of endometriosis were included. NSQIP lacks information on preoperative and postoperative patient symptoms, and the severity of endometriosis which could independently increase the risk of complications. NSQIP also only contains information on complications that happen within 30 days of surgery. Complications such as cuff cellulitis or dehiscence can present after 30 days and may be left out of the database. Furthermore, whether uteri were removed by morcellation, mini-laparotomy or colpotomy could not be accounted for, nor could we account for conventional vs. robotic laparoscopy. Removal of the uterus through a posterior colpotomy may potentially increase the risk of infection compared to morcellation, as the vagina is breached [26]. Organ space surgical-site infection occurred more frequently in the TLH group (1.8%) than the LSH group (0.9%), though not statistically significant. Although the overall sample size was large, the vast majority of hysterectomies in this cohort included removal of the cervix. It is possible that, with a larger sample of LSCH subjects, differences observed could be amplified or diminished.

In conclusion, LSCH is associated with lower odds of total and minor complications in women with endometriosis, as compared to TLH. These findings can assist in preoperative counseling and shared decision-making with patients. The risk of long-term complications and endometriosis recurrence following LSCH should be further studied, as well as patient satisfaction and attitudes toward retaining the cervix when definitive management with hysterectomy for endometriosis is desired.

## Supplementary Information

Below is the link to the electronic supplementary material.

Supplementary file1 (DOCX 13 KB)

## Data Availability

The data that support the findings of this study are available on request from the corresponding author.

## Abbreviations

*TLH*: Total laparoscopic hysterectomy
LSCH: Laparoscopic supracervical hysterectomy
*NSQIP*: National surgical quality improvement program
*CPT*: Current procedural terminology
*ASA*: American society of anesthesiologists
