# Supplementary material for: Postoperative outcomes in minimally invasive total versus supracervical hysterectomy for endometriosis: a NSQIP study
Source: Arch Gynecol Obstet. 2024 Oct 16;311(3):757–63. doi: 10.1007/s00404-024-07749-y (PMC11920324; doi:10.1007/s00404-024-07749-y)
Supplement: Supplementary file 1 — Supplementary file1 (DOCX 13 KB) [file 404_2024_7749_MOESM1_ESM.docx]

**Table S1**. Current Procedural Terminology (CPT) Codes for Hysterectomy Type

58570, 58571, 58572, 58573: Total Laparoscopic Hysterectomy
58541, 58542, 58543, 58544: Laparoscopic Supracervical Hysterectomy

**Table S2**. International Classification Diagnosis (ICD) Codes for Endometriosis

| ICD-9 Codes 617.1 Endometriosis of ovary 617.2 Endometriosis of fallopian tube 617.3 Endometriosis of pelvic peritoneum 617.4 Endometriosis of rectovaginal septum and vagina 617.5 Endometriosis of intestine 617.8 Endometriosis of other specified sites 617.9 Endometriosis, site unspecified |
| --- |
| ICD-10 Codes N80 Endometriosis N80.0 Endometriosis of uterus N80.1 Endometriosis of ovary N80.2 Endometriosis of fallopian tube N80.3 Endometriosis of pelvic peritoneum N80.4 Endometriosis of rectovaginal septum and vagina N80.5 Endometriosis of intestine N80.8 Other endometriosis N80.9 Endometriosis, unspecified |
